# Prostacyclin, thromboxane and glomerular filtration rate are abnormal in sickle cell pregnancy

**DOI:** 10.1371/journal.pone.0184345

**Published:** 2017-09-07

**Authors:** Opeyemi Abayomi Obilade, Alani Suleimon Akanmu, Fiona Broughton Pipkin, Bosede Bukola Afolabi

**Affiliations:** 1 Department of Obstetrics and Gynaecology, Lagos University Teaching Hospital, Idi-Araba, Lagos, Nigeria; 2 Department of Obstetrics and Gynaecology, State House Medical Centre, Aso Rock, Asokoro, Abuja, Nigeria; 3 Department of Haematology and Blood Transfusion, College of Medicine, University of Lagos, Idi-Araba, Lagos, Nigeria; 4 Department of Haematology and Blood Transfusion, Lagos University Teaching Hospital, Idi-Araba, Lagos, Nigeria; 5 Department of Obstetrics and Gynaecology, Faculty of Medicine and Health Sciences, University of Nottingham, Nottingham, United Kingdom; 6 Department of Obstetrics and Gynaecology, College of Medicine University of Lagos, Idi-Araba, Lagos, Nigeria; Boston University, UNITED STATES

## Abstract

**Background:**

Pregnancy increases the risk of morbidity and mortality in sickle cell disease. We previously showed pregnant women with sickle cell disease to have a relatively low plasma renin concentration in late pregnancy, associated with a lack of the expected plasma volume expansion. We hypothesized this to be due to increased systemic vascular resistance through an imbalance between the vasodilator prostacyclin and vasoconstrictor thromboxane, associated with decreased glomerular filtration rate (GFR).

**Objective:**

To compare estimated prostacyclin, thromboxane and GFR in non-pregnant and pregnant women with hemoglobin SS (HbSS) and AA (HbAA).

**Study design:**

Four groups of 20 normotensive, nulliparous women were studied in Lagos, Nigeria: pregnant HbSS or HbAA women at 36–40 weeks gestation; non-pregnant HbSS and HbAA controls. We measured stable metabolites of prostacyclin and thromboxane A_2_ by enzyme-linked immunosorbent assay; GFR using the Cockcroft-Gault equation. Data analysis was by independent (Student’s) *t*-test or Mann-Whitney U test for comparisons between any two groups of continuous variables, univariate ANOVA for multiple groups and Pearson’s correlation coefficient for degree of association between variables.

**Results:**

HbSS women had lower serum 6-keto-PGF1α concentrations than HbAA, whether pregnant or non-pregnant (P<0.001; P<0.004 respectively). Conversely, pregnant HbSS women had higher serum TxB2 (P<0.001); non-pregnant HbSS women had non-significantly higher TxB2 concentrations. The 6-keto-PGF1α:TxB2 ratio was markedly increased (pro-vasodilatory) in HbAA pregnancy (P<0.001) but reduced in HbSS pregnancy (P = 0.037). GFRs (mL/min) were higher in non-pregnant HbSS than HbAA (P<0.008) but only marginally raised in HbSS women in late pregnancy (P = 0.019) while markedly raised in HbAA pregnancy (P<0.001).

**Conclusion:**

The lower ratio of prostacyclin-thromboxane metabolites in HbSS pregnancy may indicate endothelial damage and an increased tendency to vasoconstriction and clotting. If confirmed by subsequent longitudinal studies, interventions to increase prostacyclin and reduce thromboxane, such as low dose aspirin, may be potentially useful in their management.

## Introduction

Sickle cell disease is a hemoglobinopathy that presents with various complications due to chronic anemia, hemolysis and occlusion of small vessels [1, 2]. Homozygous Hemoglobin SS is the most severe form [1, 3]. Pregnant women with sickle cell disease have a high incidence of mortality and of morbidity in the form of infections and vaso-occlusive presentations or “crises’. Their babies are also more likely to be of low birth weight and have a higher likelihood of perinatal mortality [4, 5].

In normal pregnancy, plasma volume (PV) rises early in the first trimester and plateaus at about 34 weeks’ gestation. This ~50% rise [6, 7] is associated with improved uteroplacental perfusion, oxygenation, and good fetomaternal outcomes [8, 9]; poor PV expansion is associated with poor outcomes such as preeclampsia and fetal growth restriction [10, 11].

Previous studies in pregnant women with sickle cell disease have reported little or no PV expansion especially in late pregnancy, compared with their non-pregnant counterparts [12, 13]. We have previously examined PV in the context of volume regulatory hormones including components of the renin-angiotensin-aldosterone system (RAAS) and found a relatively low plasma renin concentration in late pregnancy compared with early pregnancy, and compared with pregnant HbAA controls [13]. We hypothesized that pregnant HbSS women may have a generalized increase in systemic vascular resistance in late pregnancy leading to a reduction in renin synthesis or secretion and suggested that this could be due to an imbalance in vasoactive substances such as prostacyclin and thromboxane.

Prostacyclin (prostaglandin I_2_; PGI_2_), and thromboxane (thromboxane A_2_; TXA_2_) are eicosanoids with opposing actions. PGI_2_ is a very potent vasodilator and inhibitor of platelet aggregation, whereas TXA_2_ is pro-aggregatory and vasoconstrictor. In the first trimester of normal pregnancy there is an increase in vasodilatory PGI_2_ that is maintained, whilst there is a decrease in vasoconstrictive TXA_2._ There is thus a shift in the ratio towards vasodilatation and anti-aggregation, presumably contributing to the net vasodilation [14, 15]. In pregnancies which progress to pre-eclampsia, there is no rise in PGI_2_ at the end of the first trimester [16] so there is a reversal in the prostacyclin:thromboxane ratio and also an inhibition of the RAAS [15, 17].

Like PV, the glomerular filtration rate (GFR) increases significantly in pregnancy from as early as nine weeks of normal pregnancy. This is because there is a greater renal vasodilatation than the general systemic vasodilatation of pregnancy, leading to increased renal blood flow and GFR [18]. If, therefore, we expect there to be an increase in generalized vascular resistance in HbSS pregnant women, we would expect their GFR to be reduced in parallel with PV, particularly in late pregnancy. GFR is usually raised in people with sickle cell disease but begins to fall in early adulthood [19, 20]. It has been found to be reduced in preeclamptic pregnancies [21, 22], but has not previously been examined in pregnant women with HbSS. In our previous study [13], we also found that non-pregnant HbSS women had a supranormal PV that did not change in pregnancy despite the blunted renin release, and postulated renal dysfunction to be a possible cause.

We therefore hypothesized that pregnant HbSS women would have a reduced GFR in late pregnancy and that there would be a reversal in prostacyclin:thromboxane ratio in them as seen in preeclampsia. We measured concentrations of the major serum metabolites of PGI_2_ and TXA_2_ as well as GFR in HbSS and HbAA pregnancies and controls to examine these hypotheses.

## Materials and methods

The study was conducted primarily at the Lagos University Teaching Hospital (LUTH), Lagos, in South West Nigeria, a not for profit hospital, owned by the Federal Government of Nigeria; limited recruitment also occurred in one teaching and seven non-teaching general hospitals in Lagos State. Ethical approval was obtained from the health research and ethics committees of the Lagos University Teaching Hospital (ref no. ADM/DCST/HREC/738), Lagos State University Teaching Hospital (ref no LREC/10/06/325) and the Health Services Commisssion (ref no SHMB/728/Vol.VI/57).

This was a comparative cross-sectional study that comprised four groups of women: pregnant HbSS, pregnant HbAA, non-pregnant HbSS, and non-pregnant, healthy HbAA volunteers. They were clinically stable, normotensive, non-smoking, nulliparous women aged between 18 and 35 years with no use of aspirin or non-steroidal anti-inflammatory drugs within the preceding month and no history of hypertension, diabetes mellitus, renal disease or other chronic medical diseases. The pregnant women had singleton pregnancies between 36 and 40 completed weeks of gestation, with no emergency admission within the preceding month and no antepartum hemorrhage, suspected intrauterine growth restriction, ruptured membranes or other obstetric complication. The last menstrual period was used to determine gestational age as few women in this environment present early enough for routine first trimester scans. The HbSS women (pregnant and non-pregnant) were in a stable state with no crises or blood transfusion in the preceding 4 weeks.

The study was carried out between November 2012 and March 2014. Consecutive HbSS pregnant woman who met the inclusion criteria were invited to participate. Once written, informed consent had been obtained, the next attending HbAA pregnant woman matched for age and gestational age was also recruited, having given consent. The non-pregnant HbSS women were recruited from the Sickle Cell clinic of LUTH. The non-pregnant HbAA women were healthy staff and student volunteers from LUTH who were also age and parity matched (Table 1).

**Table 1:** Baseline characteristics of the women.

Groups N = 20 each				
Characteristic	PrSS	PrAA	NPSS	NPAA
Age (years)	26.6 ± 3.4	28.4 ± 3.1	23.6 ± 5.1	25.9 ± 4.2
Weight (kg)	67.0 ± 11.6	78.4 ± 15.9 *	52.5 ± 9.0	58.0 ± 10.2
Height (m)	1.64 ± 0.07	1.62 ± 0.07	1.64 ± 0.08	1.65 ± 0.06
BMI (kg/m^2^)	24.7 ± 3.5	29.9 ± 4.4 *	19.5 ± 2.7	21.4 ± 3.5
Systolic BP (mmHg)	116 ± 11	113 ± 10	108 ±11	106 ±10
Diastolic BP	67 ± 9	69 ± 5	64 ± 11	64 ± 6

N = number studied in each group. Age, body weight, height and BMI are reported as mean ± standard deviation.

* refers to comparisons of pregnant SS with pregnant AA, P<0.05. PrSS = pregnant HbSS, PrAA = pregnant HbAA, NPSS = non-pregnant HbSS and NPAA = non-pregnant HbAA women.

We calculated that 18 pregnant HbSS women and 18 age and parity matched pregnant hemoglobin AA controls would give an 80% power of achieving a difference in serum PGI_2_ concentration of at least 10% from a reported mean of 254.5 pg/ml seen in normal pregnant women at 40 weeks gestation [15] at a 5% significance level. We recruited 20 women per group including non-pregnant HbAA and HbSS and measured their heights and weights. We also measured their blood pressures whilst sitting comfortably, using mercury sphygmomanometers with the upper arm at the level of the heart. A 6 ml venous blood sample was taken; 4ml were placed in a gel clot activator vacuum tube and 2ml in a potassium-EDTA (ethylene diamine tetra-acetic acid) tube. Samples were transported to the Central Research Laboratory (CRL) in LUTH, using a thermal bag with ice packs. The potassium-EDTA whole blood samples were frozen at -20°C and the gel clot activator tube samples were immediately centrifuged and the serum stored at -80°C. The samples were pooled and analyzed in batches.

The stable serum metabolites of prostacyclin (6-keto-prostaglandin F1α) and thromboxane A_2_ (thromboxane B_2_) were measured via enzyme-linked immunosorbent assay (ELISA) using the Cusabio® kit from Flarebio Biotech, USA (http://www.cusabio.com). Serum creatinine was measured using the Jaffe method in the CRL. Whole blood hemoglobin genotyping was performed by electrophoresis for confirmation of recorded genotype also in the CRL. We used the Cockcroft-Gault equation (CG) to estimate GFR, bearing in mind its limitations [23, 24].

### Data analysis

Data entry and analysis were done using the IBM^®^ Statistical Package for the Social Sciences (SPSS) version 23. Data were tested for normality of distribution using the frequency distribution analysis. If they were not normally distributed, they were normalized by logarithmic transformation or non-parametric tests were used as necessary. The location and spread of continuous variables were described by the mean and standard deviation (SD) respectively (if normal in distribution) or by the median (M) and inter-quartile range respectively (skewed distribution). They are presented in the format—mean ±SD or, M (25^th^ centile, 75^th^ centile) as appropriate. Hypothesis testing for comparisons between any two groups of continuous variables was done using the independent (Student’s) *t*-test (normal distribution) or the Mann-Whitney U test (skewed data). Multiple groups of continuous variables were compared using univariate Analysis of Variance (ANOVA). Pearson’s correlation coefficient was used for measurement of the degree of association between variables. The null hypothesis was rejected at P<0.05.

## Results

All four groups were similar in age and height but the HbSS women had lower mean weights and BMI as expected and the difference was statistically significant in the pregnant women (Table 1). Thirty-nine of the 40 pregnant women studied were between 36 and 38 weeks gestation while the 40^th^ woman was 40 weeks pregnant. All the women studied were normotensive and the mean systolic and diastolic blood pressures of the pregnant HbSS women did not differ significantly from the pregnant HbAA women; p = 0.30 and 0.48 for systolic and diastolic comparisons respectively (Table 1). The women were followed up for an average of 14.7 ± 9.0 days from recruitment to delivery being 16.2 ± 9.5 days for the HbAA women and 13.2 ± 8.6 days for the HbSS women (p = 0.31).

### Eicosanoids (Table 2; Fig 1)

#### Genotype comparison

HbSS women had significantly lower serum 6-keto-prostaglandin F1α (6-keto-PGF1α) concentrations than HbAA women both in the pregnant and non-pregnant states (p = 0.019 and p = 0.004 respectively). They also had higher serum TxB2 concentrations in pregnancy than HbAA women (p<0.001). For prostacyclin:thromboxane ratio, HbSS women had significantly lower median prostacyclin:thromboxane ratios than their HbAA counterparts and this was particularly marked when comparing the pregnant women in whom prostacyclin:thromboxane ratio was almost 3 fold lower than that of pregnant HbAA (p<0.001).

**Fig 1:**
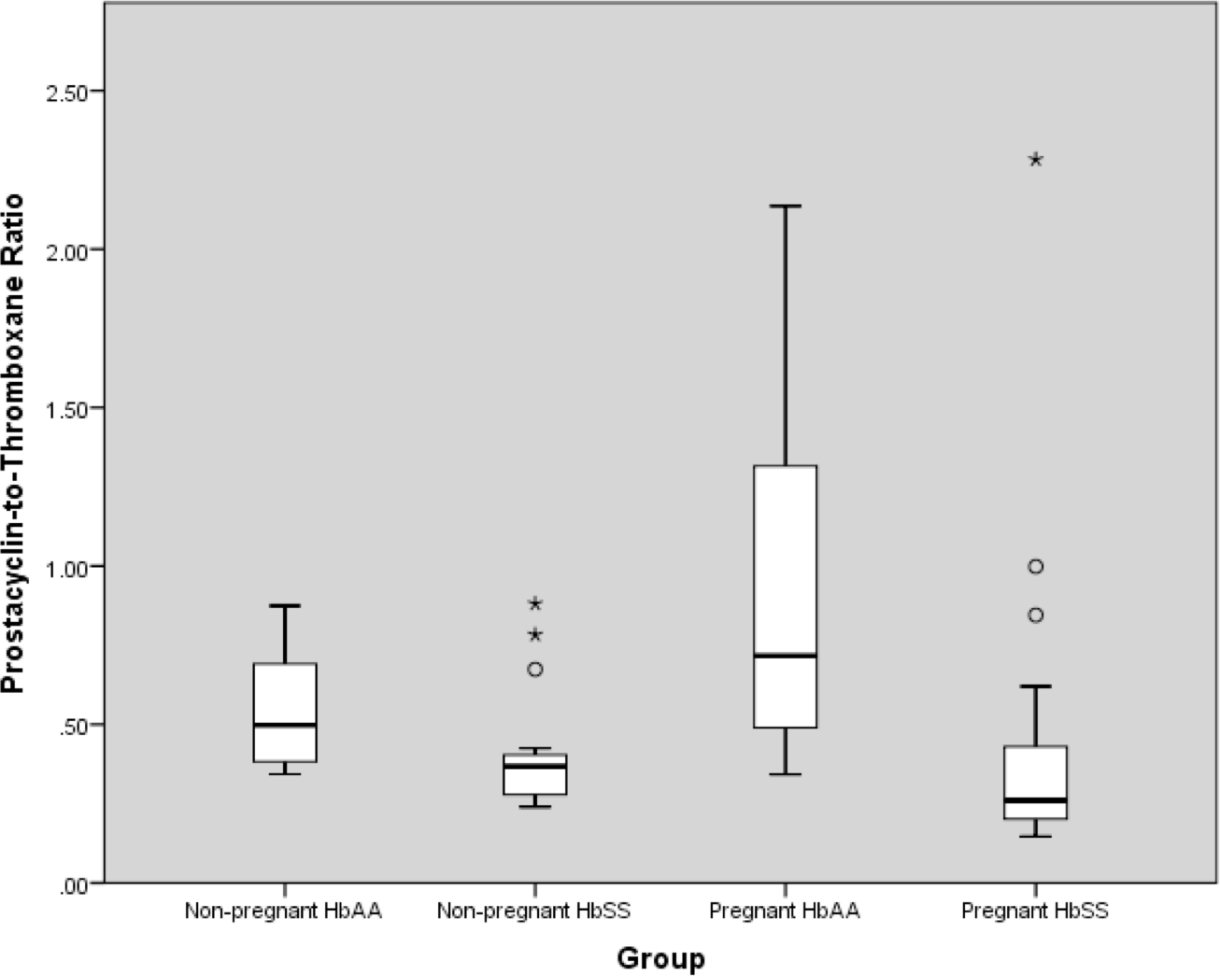
Prostacyclin-to-thromboxane ratio in pregnant and non-pregnant HbSS and HbAA women.

**Table 2:** Eicosanoids, creatinine and GFR in the groups studied.

Parameters		Groups (n = 20 in each group)^#^												
		NPAA vs NPSS			NPAA vs PrAA			NPSS vs PrSS			PrAA vs PrSS		
		NPAA	NPSS	*p*	NPAA	PrAA	*p*	NPSS	PrSS	*p*	PrAA	PrSS	*p*
**6kPGF1**α **(pg/mL)**	Mean	208.75	174.35	**0.004**	208.75	132.71	**<0.001**	174.35	105.62	**<0.001**	132.71	105.62	**0.019**
	SD	47.09	45.61		47.09	22.40		45.61	20.44		22.40	20.44	
**TxB2** **(pg/mL)**	Mean	419.59	473.80	**0.173**	419.59	203.84	**<0.001**	473.80	370.05	**0.010**	203.84	370.05	**<0.001**
	SD	85.38	125.34		85.38	111.59		125.34	163.23		111.59	163.23	
**Prostacyclin-** **thromboxane** **ratio**	Median	0.498	0.368	**0.008**	0.498	0.717	**0.044**	0.368	0.261	**0.037**	0.717	0.261	**<0.001**
	IQR	0.378, 0.693	0.278, 0.409		0.378, 0.693	0.481, 1.339		0.278, 0.409	0.199, 0.462		0.481, 1.339	0.199, 0.462	
**Creatinine (μmol/L)**	Mean	69.76	54.90	**0.002**	69.76	56.08	**0.003**	54.90	57.94	**0.503**	56.08	57.94	**0.681**
	SD	7.86	21.73		7.86	8.94		21.73	14.19		8.94	14.19	
**GFR (mL/min)**	Mean	99.23	130.24	**0.008**	99.23	165.23	**<0.001**	130.24	145.42	**0.186**	165.23	145.42	**0.085**
	SD	15.48	42.57		15.48	35.20		42.57	43.33		35.20	43.33	

#: n = 20 in each group except for 6-ketoPGF1α in the pregnant HbSS women in which two samples were inadvertently omitted during analysis thus prostacyclin (and prostacyclin:thromboxane ratio) for that group had n = 18. The other groups were unaffected and thromboxane on its own was unaffected. 6kPGF1α = prostacyclin metabolite, TxB2 = thromboxane metabolite. PrSS = pregnant HbSS, PrAA = pregnant HbAA, NPSS = non-pregnant HbSS and NPAA = non-pregnant HbAA women.

#### Pregnant versus non-pregnant

Pregnant HbAA women had significantly lower serum 6-keto-PGF1α and serum thromboxane B concentrations than the non-pregnant HbAA (p<0.001 in both cases). However, the proportional reduction in serum TxB2 concentration was much greater than that in 6-keto-PGF1α and as such they had a significantly higher prostacyclin:thromboxane ratio than their non-pregnant counterparts as expected (p<0.044). In pregnant HbSS women, although both serum 6-keto-PGF1α and TxB2 were significantly lower than in non-pregnant HbSS (p<0.001 and p = 0.010 respectively), the serum TxB2 was sufficiently high in the pregnant HbSS women to result in a significantly lower prostacyclin:thromboxane ratio (p = 0.037).

### Serum creatinine and GFR

ANOVA of the measured serum creatinine and calculated GFR (Table 2) showed that, in non-pregnant women, those with HbSS had significantly lower serum creatinine and higher GFR than HbAA controls (P<0.002, P<0.008 respectively). However, while pregnancy was associated with a highly significant fall in creatinine and rise in GFR in HbAA women (P<0.003, P<0.0001), neither occurred in HbSS women (P>0.5, P = 0.186 respectively).

### Relationship between eicosanoids and GFR

**Fig 2:**
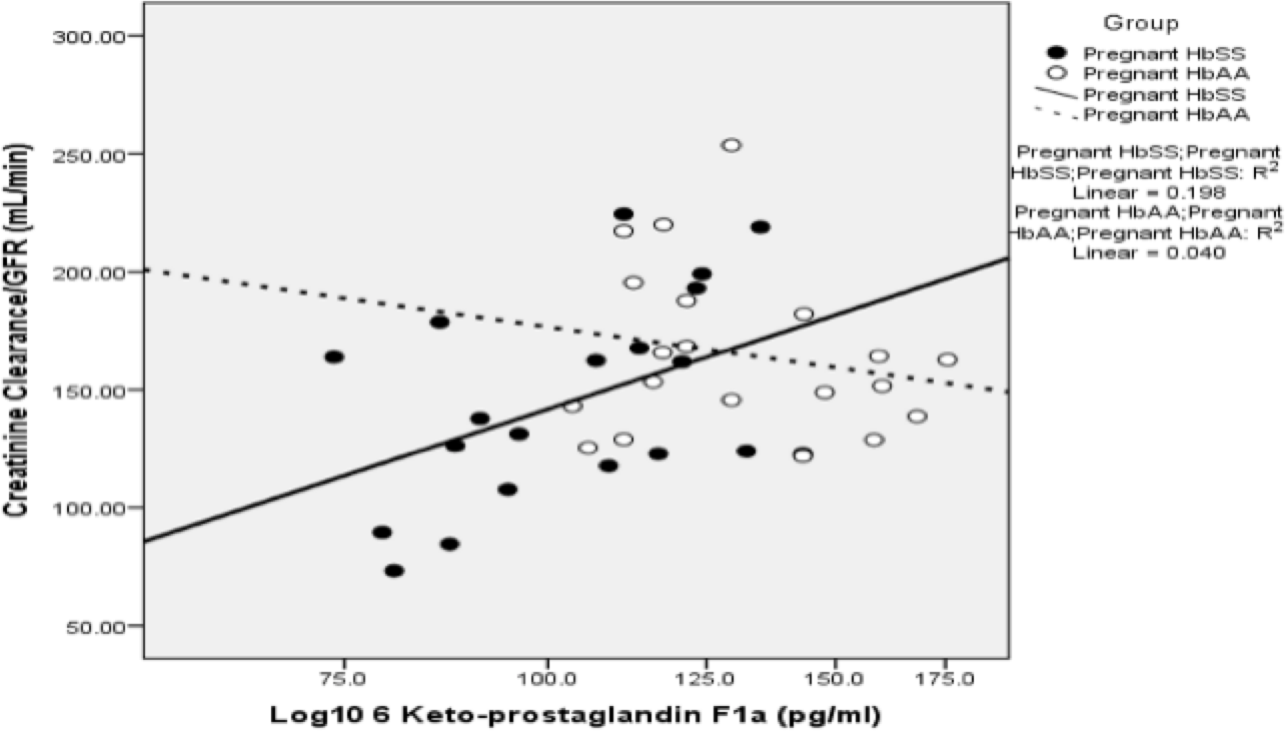
Relationship between prostacyclin and GFR. A scatter plot showing the relationship between log10 6-keto-PGF1α and estimated creatinine clearance in pregnant HbAA women (N = 20), and HbSS (N = 18) women. The computed best line of fit for both groups of women is displayed and there is a significant positive correlation in HbSS women as shown.

There was a positive correlation between log_10_ 6-keto-PGF1α and GFR (r = 0.445, p = 0.049) in HbSS pregnant women (Fig 2), but not in pregnant HbAA (P>0.3) or in either group outside pregnancy. Further exploration of possible determinants of GFR in pregnant HbSS women using univariate ANOVA identified significant effects of 6-keto-PGF1α (P<0.001), diastolic pressure (P = 0.004) and age (P = 0.012). This was confirmed by regression analysis (final r = 0.794, adjusted r^2^ = 0.561; P = 0.001). There were no other significant correlations between GFR and the eicosanoids.

### Pregnancy outcome

All physiological studies in pregnancy are ultimately aimed at identifying determinants of successful outcome for both mother and baby. The gestational age at delivery of the HbSS women (n = 19) was significantly lower than that of the HbAA women (n = 20; 38.9 ± 0.9 vs. 39.9 ± 1.0 weeks, p = 0.002). Two of the HbSS women died, one undelivered due to anemia and cardiopulmonary failure in the second stage of labor, while the second maternal death was due to sepsis and post-partum eclampsia that occurred 24 hours after her delivery by caesarean section. These women had two of the three lowest serum 6-keto-PGF1α concentrations in the entire study; the remaining very low 6-keto-PGF1α was also found in a pregnant woman with HbSS. There were no maternal mortalities in the HbAA women but there was one intrauterine fetal death from severe preeclampsia. The mean birth weight of the babies live born to the HbSS women (n = 17) was significantly lower than that of the HbAA (n = 20; 2.83 ± 0.30 vs. 3.35 ± 0.46 kg, p<0.001). Three of the HbSS babies died (2 still births, one early neonatal death).

Birth weight was correlated with GFR in HbAA women (r = 0.504, p = 0.024), but not in HbSS women (P>0.8). There were no significant correlations between birthweight and any of the eicosanoids in either group of women.

## Discussion

This is, to our knowledge, the first study of eicosanoids and renal function in women with sickle cell disease, and revealed significant differences in both pregnant and non-pregnant HbSS women by comparison with HbAA controls. Serum 6-keto-PGF1α, the major stable metabolite of PGI_2_, was lower, and thromboxane B2, higher in both groups of HbSS women than in the HbAA controls. Pregnant women with sickle cell disease had a markedly (2.7 fold) lower prostacyclin:thromboxane ratio than pregnant HbAA women, which supported our hypothesis. They also had a lower prostacyclin:thromboxane ratio than non-pregnant HbSS women and pregnant HbAA controls. This is in spite of having similar systolic and diastolic blood pressures and implies that they may be in a state of relative vasoconstriction compared to their HbAA counterparts as well as compared to the non-pregnant state. In addition, we found that they do not have a significant increase in GFR in pregnancy compared to the non-pregnant state, unlike HbAA pregnant women who do. A significant rise in GFR during pregnancy has been repeatedly shown in other populations, beginning early in the first trimester [18].

The consistently lower serum 6-keto-PGF1α and higher TxB2 concentrations in HbSS women accord with the vascular endothelial damage and platelet activation known to occur in sickle cell disease [25]. A relatively raised TXA_2_ concentration compared to PGI_2_ in the pregnant HbSS women implies a likelihood of vasoconstriction with subsequent maternal hypertension and impaired uteroplacental blood flow, as well as platelet aggregation and clotting [26].

This is interesting as the main pathologies that are frequently described in sickle cell disease are related to vaso-occlusion and hemolysis, with vaso-occlusion previously appearing to cause more of the complications. It has been shown that hemolysis results in nitric oxide depletion, which leads to endothelial dysfunction and vasoconstriction and this is thought to be the mechanism behind such sickle related pathologies as pulmonary hypertension, stroke and chronic leg ulcers [1, 27].

The pathophysiology of preeclampsia also culminates in endothelial dysfunction and vasoconstriction. Moreover, a reduced prostacyclin:thromboxane ratio has been described in preeclampsia and is the basis for the use of low dose aspirin for prevention in pregnant women known to be at high risk of preeclampsia [28, 29]. Despite previous conflicting results from individual studies about the association of preeclampsia with sickle cell disease, recent meta-analyses have confirmed a higher risk of preeclampsia in pregnant women with sickle cell disease [4, 30]. It is therefore plausible that the use of low dose aspirin may reduce the risk of preeclampsia and other complications such as IUGR in pregnant women with sickle cell disease.

An unexpected finding was that the metabolites of PGI_2_ and TXA_2_ measured in this study were both found to be lower in normal pregnancy than in the non-pregnant, albeit with the maintenance of the expected increase in prostacyclin:thromboxane ratio. Most published studies have been carried out on Caucasian women or those living in temperate climates, and have reported higher PGI_2_ and TXA_2_ concentrations in pregnancy [26, 31]. This difference may be due to the tropical hot climate in our setting and the possibility that our women are already significantly vasodilated before beginning pregnancy.

With respect to the renal function, serum creatinine concentration fell and GFR was elevated in the pregnant HbAA women, compared with the non-pregnant AA as expected [6, 18]. This pregnancy-related rise in GFR was small and not statistically significant in HbSS pregnancy, and was in fact less than 25% of the GFR increase in the HbAA pregnancy (Table 2). The finding of significant blunting of the pregnancy-associated GFR increase in the pregnant HbSS, relative to HbAA pregnancy supports our previous finding of reduced PV expansion in pregnant HbSS women [13] since plasma volume and GFR tend to change in the same direction in pregnancy [22]. It also supports our hypothesis of a relative renal vasoconstriction and a reversal of the normal pregnancy vasodilatation in them.

Our finding of interactions between PGI_2_, diastolic blood pressure and age with GFR in the pregnant HbSS women invites further exploration of the role of PGI_2_ in the hemodynamics of pregnant women with SCD. Exogenous PGI_2_ is known to increase renal plasma flow in healthy male volunteers [32], but no detailed studies appear to have been performed in women of childbearing age.

Like plasma volume, GFR has been found to correlate positively with birthweight [33] and we found this trend in the babies of HbAA women but not in those of HbSS. This may relate to the link between renal function and increased plasma volume in pregnancy, but there are likely to be many other factors involved in birthweight determination in HbSS pregnancies, which would require specifically designed studies to unravel. Their pregnancy outcome was poor as expected with a significantly lower mean birthweight and estimated gestational age than their HbAA counterparts, as well as two maternal deaths in the HbSS women.

Prospective studies of pregnant women with sickle cell disease are uncommon and we painstakingly ensured the selection of nulliparous women in all the groups studied and got almost complete data in all parameters in the four groups studied. The study has potentially useful clinical applications with respect to the management of SCD in pregnancy, given the effects of low-dose aspirin on TXA_2_, but not PGI_2_, synthesis [34, 35]. Longitudinal studies are difficult in our environment, where we have a lot of poverty and infrastructural challenges, including communication, electricity and transportation. From the results of this initial study, we intend to follow up with studies of more detailed measurements at different points during pregnancy. If subsequent longitudinal studies examining these eicosanoids confirm our current findings, a pilot clinical trial of the effect of aspirin in HbSS pregnancy may be of potential benefit.

Another potentially useful form of treatment if our finding of thromboxane-prostacyclin ratio reversal were confirmed, would be dual thromboxane receptor antagonists and thromboxane synthase inhibitors. The advantages of these drugs would be to block all deleterious effects of other thromboxane receptor agonists apart from enzymatically formed thromboxane such as isoprostanes, as well as the total avoidance of action on other prostaglandins produced by the cyclo-oxygenase pathway such as prostacyclin [36]. However, they are still undergoing experimental trials and have not yet been approved for clinical use even outside pregnancy.

Due to the stringent selection criteria, our findings cannot be said to hold with certainty in multiparous women or those with other types of SCD such as hemoglobin SC. However, as women with hemoglobin SS are known to suffer the highest number of complications and are also the more common SCD subtype, we felt it to be more appropriate to study them. The limitation from the use of the Cockcroft Gault formula for GFR calculation in pregnancy is also noteworthy. However, we found an increase in GFR in non-pregnant women with sickle cell disorder as has been found in previous studies using the more direct methods of GFR calculation, which lends credence to our results in this study.

We conclude that the serum PGI_2_, prostacyclin:thromboxane ratio and the pregnancy-related increase in GFR are significantly lower and TXA_2_ significantly higher, in pregnant HbSS women compared with HbAA. This is similar to what is known to happen in abnormal pregnancies such as preeclampsia and unexplained IUGR. As low dose aspirin has been found to reduce the incidence of preeclampsia in high-risk pregnancy, pilot studies investigating the use of aspirin in women with sickle cell disorder and fetomaternal outcome may become feasible if our current findings can be substantiated.

## Supporting information

S1 Study data This is the study data set. (XLSX)
